# Supplementary material for: COVID-19-associated ARDS treated with DEXamethasone (CoDEX): study design and rationale for a randomized trial
Source: Rev Bras Ter Intensiva. 2020 Jul-Sep;32(3):354–62. doi: 10.5935/0103-507X.20200063 (PMC7595716; doi:10.5935/0103-507X.20200063)
Supplement: Supplementary file 1 [file rbti-32-03-0354-suppl01.pdf]

COVID-19-associated ARDS treated with DEXamethasone (CoDEX): study design and rationale for a randomized trial

Síndrome do desconforto respiratório agudo associada à COVID-19 tratada com DEXametasona (CoDEX): delineamento e justificativa de um estudo randomizado

Bruno Martins Tomazini[1,2], Israel Silva Maia[3,4], Flavia Regina Bueno[1], Maria Vitoria Aparecida Oliveira Silva[1], Franca Pellison Baldassare[1], Eduardo Leite Vieira Costa[1], Ricardo Antonio Bonifácio Moura[1], Michele Ouriques Honorato[1], André Nathan Costa[1], Alexandre Biasi Cavalcanti[3,4], Regis Goulart Rosa[4,5], Álvaro Avezum[6], Viviane Cordeiro Veiga[7], Renato Delascio Lopes[8,9], Lucas Petri Damiani[3], Flávia Ribeiro Machado[4,10], Otavio Berwanger[11], Luciano César Pontes de Azevedo[1,2,4] for the COALITION COVID-19 Brazil III Investigators

Appendix 1 - SPIRIT 2013 checklist

Section/item	Item	Description	Page
**Administrative information**			
Title	1	Descriptive title identifying the study design, population, interventions, and, if applicable, trial acronym	1
Trial registration	2a	Trial identifier and registry name. If not yet registered, name of intended registry	4
	2b	All items from the World Health Organization Trial Registration Data Set	4
Protocol version	3	Date and version identifier	6
Funding	4	Sources and types of financial, material, and other support	1
Roles and responsibilities	5a	Names, affiliations, and roles of protocol contributors	1,33
	5b	Name and contact information for the trial sponsor	1
	5c	Role of study sponsor and funders, if any, in study design; collection, management, analysis, and interpretation of data; writing of the report; and the decision to submit the report for publication, including whether they will have ultimate authority over any of these activities	1
	5d	Composition, roles, and responsibilities of the coordinating centre, steering committee, endpoint adjudication committee, data management team, and other individuals or groups overseeing the trial, if applicable (see Item 21a for data monitoring committee)	16,17
**Introduction**			
Background and rationale	6a	Description of research question and justification for undertaking the trial, including summary of relevant studies (published and unpublished) examining benefits and harms for each intervention	5,6
	6b	Explanation for choice of comparators	5
Objectives	7	Specific objectives or hypotheses	6,7
Trial design	8	Description of trial design including type of trial (eg, parallel group, crossover, factorial, single group), allocation ratio, and framework (eg, superiority, equivalence, noninferiority, exploratory)	6,9
**Methods: Participants, interventions, and outcomes**			
Study setting	9	Description of study settings (eg, community clinic, academic hospital) and list of countries where data will be collected. Reference to where list of study sites can be obtained	6
Eligibility criteria	10	Inclusion and exclusion criteria for participants. If applicable, eligibility criteria for study centres and individuals who will perform the interventions (eg, surgeons, psychotherapists)	7,8,9
Interventions	11a	Interventions for each group with sufficient detail to allow replication, including how and when they will be administered	10
	11b	Criteria for discontinuing or modifying allocated interventions for a given trial participant (eg, drug dose change in response to harms, participant request, or improving/worsening disease)	11,12
	11c	Strategies to improve adherence to intervention protocols, and any procedures for monitoring adherence (eg, drug tablet return, laboratory tests)	10,11,16,17
	11d	Relevant concomitant care and interventions that are permitted or prohibited during the trial	10,11,12
Outcomes	12	Primary, secondary, and other outcomes, including the specific measurement variable (eg, systolic blood pressure), analysis metric (eg, change from baseline, final value, time to event), method of aggregation (eg, median, proportion), and time point for each outcome. Explanation of the clinical relevance of chosen efficacy and harm outcomes is strongly recommended	6,7,13
Participant timeline	13	Time schedule of enrolment, interventions (including any run-ins and washouts), assessments, and visits for participants. A schematic diagram is highly recommended (see Figure)	Figure 1

Sample size	14	Estimated number of participants needed to achieve study objectives and how it was determined, including clinical and statistical assumptions supporting any sample size calculations	13,14
Recruitment	15	Strategies for achieving adequate participant enrolment to reach target sample size	7,8,9
**Methods: Assignment of interventions (for controlled trials)**			
Allocation:			
Sequence generation	16a	Method of generating the allocation sequence (eg, computer-generated random numbers), and list of any factors for stratification. To reduce predictability of a random sequence, details of any planned restriction (eg, blocking) should be provided in a separate document that is unavailable to those who enrol participants or assign interventions	9
Allocation concealment mechanism	16b	Mechanism of implementing the allocation sequence (eg, central telephone; sequentially numbered, opaque, sealed envelopes), describing any steps to conceal the sequence until interventions are assigned	9
Implementation	16c	Who will generate the allocation sequence, who will enrol participants, and who will assign participants to interventions	9,10
Blinding (masking)	17a	Who will be blinded after assignment to interventions (eg, trial participants, care providers, outcome assessors, data analysts), and how	10
	17b	If blinded, circumstances under which unblinding is permissible, and procedure for revealing a participant's allocated intervention during the trial	N/A
**Methods: Data collection, management, and analysis**			
Data collection methods	18a	Plans for assessment and collection of outcome, baseline, and other trial data, including any related processes to promote data quality (eg, duplicate measurements, training of assessors) and a description of study instruments (eg, questionnaires, laboratory tests) along with their reliability and validity, if known. Reference to where data collection forms can be found, if not in the protocol	12,13,16,17
	18b	Plans to promote participant retention and complete follow-up, including list of any outcome data to be collected for participants who discontinue or deviate from intervention protocols	12,13,16,17
Data management	19	Plans for data entry, coding, security, and storage, including any related processes to promote data quality (eg, double data entry; range checks for data values). Reference to where details of data management procedures can be found, if not in the protocol	12,13,16,17
Statistical methods	20a	Statistical methods for analysing primary and secondary outcomes. Reference to where other details of the statistical analysis plan can be found, if not in the protocol	14,15
	20b	Methods for any additional analyses (eg, subgroup and adjusted analyses)	15,16
	20c	Definition of analysis population relating to protocol non-adherence (eg, as randomised analysis), and any statistical methods to handle missing data (eg, multiple imputation)	14,15,16
**Methods: Monitoring**			
Data monitoring	21a	Composition of data monitoring committee (DMC); summary of its role and reporting structure; statement of whether it is independent from the sponsor and competing interests; and reference to where further details about its charter can be found, if not in the protocol. Alternatively, an explanation of why a DMC is not needed	16,17
	21b	Description of any interim analyses and stopping guidelines, including who will have access to these interim results and make the final decision to terminate the trial	14
Harms	22	Plans for collecting, assessing, reporting, and managing solicited and spontaneously reported adverse events and other unintended effects of trial interventions or trial conduct	11
Auditing	23	Frequency and procedures for auditing trial conduct, if any, and whether the process will be independent from investigators and the sponsor	NA
**Ethics and dissemination**			
Research ethics approval	24	Plans for seeking research ethics committee/institutional review board (REC/IRB) approval	17,18
Protocol amendments	25	Plans for communicating important protocol modifications (eg, changes to eligibility criteria, outcomes, analyses) to relevant parties (eg, investigators, REC/IRBs, trial participants, trial registries, journals, regulators)	17,18
Consent or assent	26a	Who will obtain informed consent or assent from potential trial participants or authorized surrogates, and how (see Item 32)	17
	26b	Additional consent provisions for collection and use of participant data and biological specimens in ancillary studies, if applicable	NA
Confidentiality	27	How personal information about potential and enrolled participants will be collected, shared, and maintained in order to protect confidentiality before, during, and after the trial	12,13

Declaration of interests	28	Financial and other competing interests for principal investigators for the overall trial and each study site	19
Access to data	29	Statement of who will have access to the final trial dataset, and disclosure of contractual agreements that limit such access for investigators	13
Ancillary and post-trial care	30	Provisions, if any, for ancillary and post-trial care, and for compensation to those who suffer harm from trial participation	NA
Dissemination policy	31a	Plans for investigators and sponsor to communicate trial results to participants, healthcare professionals, the public, and other relevant groups (eg, via publication, reporting in results databases, or other data sharing arrangements), including any publication restrictions	17,18
	31b	Authorship eligibility guidelines and any intended use of professional writers	18
	31c	Plans, if any, for granting public access to the full protocol, participant-level dataset, and statistical code	13,17,18
**Appendices**			
Informed consent materials	32	Model consent form and other related documentation given to participants and authorized surrogates	NA, per Brazilian law
Biological specimens	33	Plans for collection, laboratory evaluation, and storage of biological specimens for genetic or molecular analysis in the current trial and for future use in ancillary studies, if applicable	NA

Appendix 2 - Steering committee

COALITION COVID-19 Brazil III Investigators

Bruno Martins Tomazini, Israel Silva Maia, Eduardo Leite Vieira Costa, Alexandre Biasi Cavalcanti, Regis Goulart Rosa, Álvaro Avezum, Viviane Cordeiro Veiga, Renato Delascio Lopes, Lucas Petri Damiani, Flávia Ribeiro Machado, Otavio Berwanger, Luciano César Pontes de Azevedo for the COALITION COVID-19 Brazil III Investigators.

Appendix 3 - Statistical Analysis Plan (SAP) Version 2.0

Sample size and power

There is a lack of reliable data available in patients with acute respiratory distress syndrome (ARDS) due to COVID-19 to allow an accurate sample size calculation. We therefore used data from a randomized controlled trial in non-COVID-19 ARDS patients,[1] a well-designed multicenter trial that is representative of ARDS outcomes in Brazil, to calculate the sample size. It was assumed a mean of ventilator-free days (VFD) at 28 days of 8 days ± 9 days (standard deviation) in the control group. With a two-sided type I error of 0.05 and power of 80% to identify a difference in three days free of mechanical ventilation between groups, a sample size of 290 patients would be needed. However, in the end of May 2020, before the first interim analysis, after discussing the protocol with the Data Monitoring Committee (DMC), the Steering Committee decided to increase the sample size based on the following rational: Given the uncertainty regarding the normality of distribution of VFD, based on the Pitman Asymptotic Relative Efficiency,[2] the sample size should be increased by 15% to preserve study power coupled with a 4% increase considering possible lost to follow-up and withdrawal of consent. Therefore, a final sample size of 350 patients is needed.

Also, due to the lack of data about ventilator free days in COVID-19 patients, the sample size will be updated using the pooled standard deviation of ventilator free days of the first interim analysis, unless by the time of the first interim analysis all patients have been recruited.

The minimal clinically important difference of three days for VFD was chosen based on other trials[3,4] along with what is perceived as a significant improvement to the in-hospital complications, costs, and intensive care unit availability, especially in countries with limited resources.

Interim analysis

Two interim analyses are planned for safety and efficacy evaluation, after 96 patients and 234 patients with the complete follow up to the primary outcome. Since the recruitment rate for the study is expected to increase giving the increase in number of cases of COVID-19 in Brazil it is possible that by the time all the patients for the second interim analysis have

completed the follow-up for the primary outcome, the entire sample has already been recruited. Therefore, in this specific situation, the second interim analysis will be cancelled.

We will use the Haybittle-Peto boundary stopping rule for both safety and efficacy based on the evidence of significant differences between intervention or control group regarding ventilator free days at day 28, mortality or adverse events. The stopping rule for safety will be a p-value <0.01 and for efficacy p-value <0.001. The Haybittle-Peto boundary is a conservative stopping rule at interim analysis that has minimal impact in increasing type I error in two-arm trials.[(5)] We will not adjust the final tests for sequential analysis. The interim analyses will be performed by an external and independent DMC.

Basic reporting principles:

The baseline characteristics of the patients will be displayed as the supplementary table 1 (Table 1S):

Table 1S - Baseline characteristics of included patients

	Dexamethasone<br>n = xxx	Control<br>n = xxx
Age, mean ± SD	xx.x ± xx.x	xx.x ± xx.x
Female sex, n (%)	xx.x (xx.x)	xx.x (xx.x)
SAPS3 score, mean ± SD	xx.x (xx.x)	xx.x (xx.x)
Number of non-pulmonary organ failures, median [IQR]	x.xx [x.xx - x.xx]	x.xx [x.xx - x.xx]
Time since onset of symptoms, median [IQR], days	x.xx [x.xx - x.xx]	x.xx [x.xx - x.xx]
Days intubated prior to randomization, median [IQR]	x.xx [x.xx - x.xx]	x.xx [x.xx - x.xx]
COVID-19, n (%)		
Positive	xx.x (xx.x)	xx.x (xx.x)
Negative	xx.x (xx.x)	xx.x (xx.x)
In analysis	xx.x (xx.x)	xx.x (xx.x)
Not collected/unavailable	xx.x (xx.x)	xx.x (xx.x)
Comorbidities, n (%)		
Hypertension	xx.x (xx.x)	xx.x (xx.x)
Diabetes	xx.x (xx.x)	xx.x (xx.x)
Former smoker	xx.x (xx.x)	xx.x (xx.x)
Active smoker	xx.x (xx.x)	xx.x (xx.x)
Obesity	xx.x (xx.x)	xx.x (xx.x)
Solid tumor	xx.x (xx.x)	xx.x (xx.x)
Hematologic malignancy	xx.x (xx.x)	xx.x (xx.x)
Heart failure	xx.x (xx.x)	xx.x (xx.x)
COPD	xx.x (xx.x)	xx.x (xx.x)
AIDS	xx.x (xx.x)	xx.x (xx.x)
Chronic renal failure	xx.x (xx.x)	xx.x (xx.x)
Chronic dialysis	xx.x (xx.x)	xx.x (xx.x)
Cirrhosis	xx.x (xx.x)	xx.x (xx.x)
Asthma	xx.x (xx.x)	xx.x (xx.x)
Neuromuscular disease	xx.x (xx.x)	xx.x (xx.x)
Previous MI	xx.x (xx.x)	xx.x (xx.x)
Clinical characteristics		
Systolic BP, mmHg, mean ± SD	xx.x ± xx.x	xx.x ± xx.x
Diastolic BP, mmHg, mean ± SD	xx.x ± xx.x	xx.x ± xx.x
HR, bpm, mean ± SD	xx.x ± xx.x	xx.x ± xx.x
SpO_2, %, mean ± SD	xx.x ± xx.x	xx.x ± xx.x
HScore ≥169, N_0 (%)	xx.x (xx.x)	xx.x (xx.x)

Respiratory measures, mean ± SD		
PaO_2/FiO_2	xx.x ± xx.x	xx.x ± xx.x
Tidal volume, mL/kg predicted body weight	xx.x ± xx.x	xx.x ± xx.x
Plateau airway pressure (cmH_2O)	xx.x ± xx.x	xx.x ± xx.x
Minute ventilation, L/minute	xx.x ± xx.x	xx.x ± xx.x
Respiratory rate, breaths/minute	xx.x ± xx.x	xx.x ± xx.x
Driving pressure, cmH_2O	xx.x ± xx.x	xx.x ± xx.x
Positive end expiratory pressure, cmH_2O	xx.x ± xx.x	xx.x ± xx.x
Respiratory system static compliance, mL/cmH_2O	xx.x ± xx.x	xx.x ± xx.x
Intravenous sedation, n (%)	xx.x (xx.x)	xx.x (xx.x)
Richmond Agitation Sedation Scale, median [IQR]	x.xx [x.xx - x.xx]	x.xx [x.xx - x.xx]
Treatment during study period, n (%)		
Vasopressors	xx.x (xx.x)	xx.x (xx.x)
Renal replacement therapy	xx.x (xx.x)	xx.x (xx.x)
Use of neuromuscular blocking agents	xx.x (xx.x)	xx.x (xx.x)
ECMO	xx.x (xx.x)	xx.x (xx.x)
Prone position	xx.x (xx.x)	xx.x (xx.x)
Blood sample		
Creatinine, mg/dL, mean ± SD	xx.x ± xx.x	xx.x ± xx.x
D-dimer, ng/dL, mean ± SD	xx.x ± xx.x	xx.x ± xx.x
Hemoglobin, g/dL, mean ± SD	xx.x ± xx.x	xx.x ± xx.x
Total leucocyte count, μg/mL, median [IQR]	x.xx [x.xx - x.xx]	x.xx [x.xx - x.xx]
Platelets, $/mm^3$, mean ± SD	xx.x ± xx.x	xx.x ± xx.x
Lymphocytes, μg/mL, median [IQR]	x.xx [x.xx - x.xx]	x.xx [x.xx - x.xx]
Lactate (mg/dL)	xx.x ± xx.x	xx.x ± xx.x
Troponin, median [IQR]	x.xx [x.xx - x.xx]	x.xx [x.xx - x.xx]
Additional medication, n (%)		
Hydroxychloroquine	xx.x (xx.x)	xx.x (xx.x)
Azithromycin	xx.x (xx.x)	xx.x (xx.x)
Other antibiotics	xx.x (xx.x)	xx.x (xx.x)
Oseltamivir	xx.x (xx.x)	xx.x (xx.x)
Lopinavir+ritonavir	xx.x (xx.x)	xx.x (xx.x)
Use of corticosteroids before randomization, n (%)	xx.x (xx.x)	xx.x (xx.x)

The main analysis study population will comprise all patients who have been randomized (intention-to-treat population), using the group allocated as variable, regardless of the medication administered.

The primary objective is to evaluate the effectiveness of early intravenous (IV) dexamethasone administration in ventilator-free days at 28 days after randomization, defined as alive and free from mechanical ventilation in adult patients with moderate or severe ARDS due to confirmed or probable SARS-CoV-2 infection. Patients discharged from the hospital alive before 28 days will be considered alive and free from mechanical ventilation at day 28. Number of days free from mechanical ventilation will be presented as mean and standard deviation. The treatment effect will be presented as mean difference, with 95% confidence interval and P-value. We will use a generalized linear model with beta-binomial distribution or zero/one inflated beta distribution, with center as random effect and adjusted for age, corticosteroid use before randomization and partial pressure of oxygen/fraction of inspired oxygen (PaO_2/FiO_2) ratio.

All-cause mortality rates at 28 days will be analyzed using a mixed Cox model, with centers as random effects (frailty model). The treatment effect on Sequential Organ Failure Assessment (SOFA) score 48h, 72h, and 7 days after randomization will be analyzed by a linear mixed model with centers as random effects. For the clinical status of patients, an ordinal logistic regression will be used. The results will be presented as a proportional odds ratio comparing two combinations: Intervention versus Control. The probability ratios will be derived from a mixed logistic regression of proportional probabilities adjusted for age and PaO_2/FiO_2 ratio, with random intercepts for the center. The cumulative ordinal scores will be presented separately, as well as the main secondary results. Each odds ratio will be estimated using mixed logistic

regression. The same models will be used to compare the effects of treatment on the follow-up. In case of the proportional odds assumption is not met, categories of the Ordinal scale 1-4 will be grouped as a single category for the analysis. All secondary outcomes are exploratory and no adjustment for multiple testing will be made.

Adverse events will be expressed as counts and percentages and compared between groups using the Chi-square test. The main results will be displayed as the supplementary table 2 (Table 2S). The significance level for all analyses will be 0.05. There will be no adjustment for multiple testing. All analyses will be performed using the R software[6] (R Core Team, Vienna, Austria, 2020).

Table 2S - Main results presentation

Outcomes	Dexamethasone	Control	Treatment effect Dexamethasone *versus* Control	
	n = xxx	n = xxx	[IC95%]	p valor
Primary outcome				
Ventilator free days from 1 to 28 d, mean (SD)	xx.x ± xx.x	xx.x ± xx.x	x.xx [x.xx; x.xx]	x.xx
Secondary outcomes				
Clinical status at day 15, n (%)				
Category 1 - 5 *versus* 6 (alive *versus* dead)	x.xx (x.xx)	x.xx (x.xx)	x.xx [x.xx; x.xx]	x.xx
Category 1 - 4 *versus* 5 - 6	x.xx (x.xx)	x.xx (x.xx)	x.xx [x.xx; x.xx]	x.xx
Category 1 - 3 *versus* 4 - 6	x.xx (x.xx)	x.xx (x.xx)	x.xx [x.xx; x.xx]	x.xx
Category 1 - 2 *versus* 3 - 6	x.xx (x.xx)	x.xx (x.xx)	x.xx [x.xx; x.xx]	x.xx
Category 1 *versus* 2 to 6 (at home *versus* hospital or dead)	x.xx (x.xx)	x.xx (x.xx)	x.xx [x.xx; x.xx]	x.xx
All-cause mortality at 28 days	x.xx (x.xx)	x.xx (x.xx)	x.xx [x.xx; x.xx]	x.xx
Number of days of MV from 1 to 28 d	x.xx (x.xx)	x.xx (x.xx)	x.xx [x.xx; x.xx]	x.xx
ICU free days at 28 days	x.xx (x.xx)	x.xx (x.xx)	x.xx [x.xx; x.xx]	x.xx
SOFA scores				
48 hours	x.xx (x.xx)	x.xx (x.xx)	x.xx [x.xx; x.xx]	x.xx
72 hours	x.xx (x.xx)	x.xx (x.xx)	x.xx [x.xx; x.xx]	x.xx
7 days	x.xx (x.xx)	x.xx (x.xx)	x.xx [x.xx; x.xx]	x.xx
Adverse events				
New diagnosis of infection until day 28, n (%)	x.xx (x.xx)	x.xx (x.xx)	x.xx [x.xx; x.xx]	x.xx
Insulin use for hyperglycemia, n (%)	x.xx (x.xx)	x.xx (x.xx)	x.xx [x.xx; x.xx]	x.xx

Sensitivity analyses

We plan to perform analyses to assess treatment effects on the primary and secondary outcomes considering only patients that received the proposed treatment in the intervention group and patients that not received corticosteroids in the control group (per protocol analysis). Additionally, we will also perform sensitivity analysis for the primary outcome in the following groups:

1. Confirmed COVID-19 infection.
2. Confirmed and probable COVID-19 infection.
3. Patients which received corticosteroids and patients which did not received corticosteroids (as treated analysis).
4. Patients which received the proposed treatment in the intervention group and patients that not received corticosteroids in the control group (Per protocol analysis).

Subgroup analyses

Will also perform subgroup analysis adding an interaction parameter with group in the main model for (Table 3S):

1. Age, years (< 60 and ≥ 60).
2. PaO_2/FiO_2 ratio, mmHg (≤ 100 and >100).
3. Simplified Acute Physiology Score 3 (SAPS3), points (< 50 and ≥ 50).
4. Duration of symptoms at randomization, days (≤ 7 and > 7).

5. Duration of moderate / severe ARDS to randomization, hours (≤ 24 hours and > 24hours to 48 hours).
6. Position at randomization; (prone or supine).
7. HScore (≥ 169 and < 169).
8. Use of corticosteroids before randomization.
9. Use of vasopressors at randomization.

Finally, the ordinal score (secondary outcome) will be available daily for each patient up to 15 days. These results will be presented in an alluvial graph for each arm. The conditional probabilities of change in stages (1 to 6) will be estimated via Bayesian Networks to better describe the time when the intervention can change the distribution of the score or estimate the probability of discharge. For example, Bayesian networks allow estimating the probability of discharge on day 6 if the patient is without oxygenation support on day 5. We plan to present this independent manuscript analysis with and report transition probabilities and relative risk (with confidence intervals obtained through bootstrap techniques) for relevant scenarios.

Table 3S - Effect of dexamethasone vs control on ventilator free days according to subgroups

Subgroups	Dexamethasone	Control	Treatment effect Dexamethasone *versus* Control	
	n = xxx	n = xxx	HR [IC95%]	p valor
Age				
< 60 years	x.xx (x.xx)	x.xx (x.xx)	x.xx [x.xx; x.xx]	x.xx
≥ 60 years	x.xx (x.xx)	x.xx (x.xx)	x.xx [x.xx; x.xx]	x.xx
PaO_2/FiO_2				
≤ 100mmHg	x.xx (x.xx)	x.xx (x.xx)	x.xx [x.xx; x.xx]	x.xx
> 100mmHg	x.xx (x.xx)	x.xx (x.xx)	x.xx [x.xx; x.xx]	x.xx
SAPS 3				
< 50	x.xx (x.xx)	x.xx (x.xx)	x.xx [x.xx; x.xx]	x.xx
≥ 50	x.xx (x.xx)	x.xx (x.xx)	x.xx [x.xx; x.xx]	x.xx
Duration of symptoms at randomization, d				
≤ 7	x.xx (x.xx)	x.xx (x.xx)	x.xx [x.xx; x.xx]	x.xx
> 7	x.xx (x.xx)	x.xx (x.xx)	x.xx [x.xx; x.xx]	x.xx
Duration of moderate / severe ARDS to randomization, h				
≤ 24	x.xx (x.xx)	x.xx (x.xx)	x.xx [x.xx; x.xx]	x.xx
> 24-48	x.xx (x.xx)	x.xx (x.xx)	x.xx [x.xx; x.xx]	x.xx
Position at randomization				
Supine	x.xx (x.xx)	x.xx (x.xx)	x.xx [x.xx; x.xx]	x.xx
Prone	x.xx (x.xx)	x.xx (x.xx)	x.xx [x.xx; x.xx]	x.xx
HScore				
< 169	x.xx (x.xx)	x.xx (x.xx)	x.xx [x.xx; x.xx]	x.xx
≥ 169	x.xx (x.xx)	x.xx (x.xx)	x.xx [x.xx; x.xx]	x.xx
Use of corticosteroids before randomization				
Yes	x.xx (x.xx)	x.xx (x.xx)	x.xx [x.xx; x.xx]	x.xx
No	x.xx (x.xx)	x.xx (x.xx)	x.xx [x.xx; x.xx]	x.xx
Use of vasopressors at randomization				
Yes	x.xx (x.xx)	x.xx (x.xx)	x.xx [x.xx; x.xx]	x.xx
No	x.xx (x.xx)	x.xx (x.xx)	x.xx [x.xx; x.xx]	x.xx

Treatment adherence report

Patients in the intervention group should receive the intervention for 10 days or until intensive care unit (ICU) discharge, whichever comes first and patients in the control group should not receive corticosteroids. However, since it is an open label study, it is possible that deviations in the protocol happen. Thus, we will describe the use of study drug in all arms and use of corticosteroids in the control group until the 10th day.

REFERENCES

1. Writing Group for the Alveolar Recruitment for Acute Respiratory Distress Syndrome Trial (ART) Investigators; Cavalcanti AB, Suzumura EA, Laranjeira LN, Paisani DM, Damiani LP, Guimarães HP, et al. Effect of lung recruitment and titrated positive end-expiratory pressure (PEEP) vs low PEEP on mortality in patients with acute respiratory distress syndrome: a randomized clinical trial. JAMA. 2017;318(14):1335-45.
2. Lehmann EL. Nonparametrics: statistical methods based on ranks. Holden-Day; 1975.
3. Rice TW, Wheeler AP, Thompson BT, deBoisblanc BP, Steingrub J, Rock P; NIH NHLBI Acute Respiratory Distress Syndrome Network of Investigators. Enteral omega-3 fatty acid, gamma-linolenic acid, and antioxidant supplementation in acute lung injury. JAMA. 2011;306(14):1574-81.
4. Hodgson CL, Cooper DJ, Arabi Y, King V, Bersten A, Bihari S, et al. Maximal Recruitment Open Lung Ventilation in Acute Respiratory Distress Syndrome (PHARLAP). A Phase II, Multicenter Randomized Controlled Clinical Trial. Am J Respir Crit Care Med. 2019;200(11):1363-72.
5. Blenkinsop A, Parmar MK, Choodari-Oskooei B. Assessing the impact of efficacy stopping rules on the error rates under the multi-arm multi-stage framework. Clin Trials. 2019;16(2):132-41.
6. R Foundation for Statistical Computing. R: A language and environment for statistical computing [program]. Vienna, Austria: R Foundation for Statistical Computing; 2019.

Appendix 4 - Data Monitoring Committee (DMC) Charter Version 1.1. June 10th, 2020

Content	Charter details
**Introduction**	
Name of the trial	COVID-19-associated ARDS treated with DEXamethasone: CoDEX Trial<br>**Trial Intervention**<br>Intravenous dexamethasone
Objectives	**Primary objective**<br>1. Evaluate the effectiveness of early intravenous (IV) dexamethasone administration in ventilator-free days at 28 days after randomization in adult patients with moderate or severe ARDS due to confirmed or probable SARS-CoV-2 infection<br>**Secondary objectives**<br>1. All-cause mortality rates at 28 days after randomization<br>2. Clinical status of patients at 15 days after randomization using the 6-point Ordinal Scale<br>3. Number of days of mechanical ventilation from randomization to day 28<br>4. ICU free days at day 28<br>5. Change in the Sequential Organ Failure Assessment (SOFA) Score 48h, 72h and 7 days after randomization
Outline of scope of charter	The purpose of this document is to describe the membership, terms of reference, roles, responsibilities, authority, decision-making of the DMC for the CoDEX Trial, including the timing of meetings, methods of providing information to and from the DMC, frequency and format of meetings, statistical issues and relationships with other committees
**Roles and responsibilities**	
A broad statement of the aims of the committee	To protect and serve the CoDEX Trial patients regarding safety and to assist and advise the Academic Steering Committee to protect the validity and credibility of the CoDEX Trial<br>To safeguard the interests of the CoDEX Trial participants, assess the safety and efficacy of the interventions during the trial, and monitor the overall conduct of the CoDEX Trial
Terms of reference	The DMC should receive and review the progress and accruing data of the CoDEX Trial and provide advice on the conduct of the trial to the Academic Steering Committee<br>The DMC should inform the Academic Steering Committee if, in their view:<br>I. the results are likely to convince a broad range of clinicians, including those supporting the trial and the general clinical community, that one trial arm, or a subset of trial population, is clearly indicated or contraindicated, and there was a reasonable expectation that this new evidence would materially influence patient management
Specific roles of DMC	The DMC will assess and provide recommendations on the study protocol, DMC Charter, Statistical analysis plan and collected data and safety<br>The DMC will review the trial data after 96 patients and 234 of patients with complete follow up to the primary outcome. The review of the trial's progress will include data quality, and main endpoints (ventilator free days at 28 days and all-cause mortality at 28 days), including safety data
**Before or early in the trial**	
Whether the DMC will have input into the protocol	All potential DMC members should have sight of the protocol before agreeing to join the committee. Before recruitment begins the trial will have undergone review by the sponsor, scrutiny by other trial committees, a research ethics committee (REC), Medicines and Healthcare products Regulatory Agency (MHRA) and Health Research Authority. Therefore, if a potential DMC member has major reservations about the trial, they should report these to the Academic Steering Committee and may decide not to accept the invitation to join. DMC members should be independent and constructively critical of the ongoing trial, but also supportive of aims and methods of the trial
Whether the DMC will meet before the start of the trial	The DMC will meet early in the course of the trial, to discuss the protocol, the trial, any analysis plan, future meetings, and to have the opportunity to clarify any aspects with the Academic Steering Committee
Any issues specific to the disease under study	Issues specific to the disease under study should be described

Any specific regulatory issues	The DMC should be aware of any regulatory implications when making recommendations
Any other issues specific to the treatment under study	Issues specific to the treatment under study should be described
Whether members of the DMC will have a contract	DMC members do not formally sign a contract but formally register their assent to join the group by confirming (1) that they agree to be on the DMC and (2) that they agree with the contents of this Charter. Any competing interests should be declared at the same time. Members should complete and return the agreement and potential competing interests form
**Composition**	
Membership and size of the DMC	Membership will consist of three members, which include at least one clinician experienced in the clinical area and at least one experienced clinical statistician. Additional members experienced in clinical trials should reflect the other specialities involved in the trial. The DMC will be formed only by overseas members<br>The members should not be involved with the trial in any other way nor have competing interests that could impact on the trial. Any competing interests, both real and potential, must be declared. Although members may be able to act objectively despite such connections, complete disclosure enhances credibility. A short competing interest form should be completed and returned by the DMC members to the trial coordinating team (Agreement and potential competing interests form)<br>The members of the IDMC for this trial are:<br>(1) Professor Carol Hodgson (Chair)<br>(2) Professor Michael Bailey (Statistician)<br>(3) Professor Theodore Iwashyna
The Chair, how they are chosen and the Chair's role	The Chair should have previous experience of serving on DMCs and experience of chairing meetings and be able to facilitate and summarize discussions. The Chair will be chosen by the Academic Steering Committee and will be responsible for choosing the other two DMC members. The Chair is expected to facilitate and summarize discussions and keep copies of all reports and communications.<br>Other Chair's roles are:<br>Hold all DMC meetings and ensure that all relevant data is reviewed<br>Ensure that only DMC members are present during the analysis and deliberation of the DMC data<br>Approve written minutes of all closed sessions of the DMC meetings<br>Create and archive written minutes of all executive sessions of DMC meetings. These minutes will remain confidential only to DMC members until after the database has been blocked and the sponsor's disclosure<br>Arrange additional consultations with subject matter experts as needed
The responsibilities of the DMC statistician	The DMC statistician will be chosen by the DMC Chair and will provide independent statistical expertise<br>The statistician appointed by the DMC Chair will perform independent statistical analyses and have unlimited access to the entire study database. However, the analysis plan of the independent statistician of the DMC should follow the same principles described in the statistical analysis plan of the study
The responsibilities of the Steering Committee	The responsibilities of the Academic Steering Committee are:<br>1. Monitor the conduct of the study, as well as the collection and quality of the study data<br>2. Review scheduled DMC reports, with aggregated hidden data (i.e. all subjects, not separated by treatment group)<br>3. Provide joint review and approval of minutes of open and final sessions of the DMC data review meetings<br>4. Accept or reject DMC recommendations. The DMC shall be notified in writing of the response to any recommendations, including the reasoning in which the recommendations are not accepted<br>5. Communicate the recommendations of the DMC for changes in the conduct of the study to the researchers, who in turn communicate them to the Ethics Committees of each site and to the National Council of Ethics in Research (CONEP) and to the National Health Surveillance Agency (ANVISA). Communication on the DMC with researchers will be limited to formal requests for changes in the conduct of the study and will not include information on the conduct of DMC meetings<br>6. The implementation of changes to the protocol is in accordance with the DMC recommendations
**Relationships**	
Role of the funding source	The COVID-19 Brazil Coalition III is a partnership of academic leaders who designed a study initiated by a double-sponsored researcher, the Coalition in conjunction with Aché Pharmaceuticals which provided the study drug, the drug logistics distribution to the study centres and insurance for the study patients. However, Aché will have no participation or interfere with the trial design, enrolment, analysis, manuscript writing, or publication, it will have no role in the DMC´s choices, in appointing members, or in the design of this regulation. The decision to interrupt or continue the trial on the recommendation of the DMC will be entirely the responsibility of the Academic Steering Committee, without influence or supervision of the Aché. However, Aché will be notified of any decision of the steering committee as soon as the decision is made
Payments to DMC members	Each DMC member will receive a payment of US$ 1000 (One thousand United States Dollars)<br>DMC members should not use interim results to inform trading in pharmaceutical shares, and careful consideration should be given to trading in stock of companies with competing products
**Organization of DMC meetings**	
Expected frequency of DMC meetings	DMC members will meet once at the beginning of the study, at each interim analysis and whenever they deem it necessary or at the request of the Academic Steering committee, especially when new evidence emerges about the therapy being studied or when adverse events are reported

Whether meetings will be face-to-face or by teleconference	All meetings will be held by videoconference
How DMC meetings will be organized, especially regarding open and closed sessions, including who will be present in each session	The meetings will consist of open and closed parties. During the initial open part of the meeting, a steering committee member will conduct a brief presentation related to the status of the trial, its conduct, and any concerns, and will be available for questions from DMC members. The closed session, which will take place immediately after, and will be attended only by DMC members
**Trial documentation and procedures to ensure confidentiality and proper communication**	
Confidentiality regarding trial's information	To protect the scientific integrity of the study under review, all members of the DMC agree to keep all information in absolute secrecy and will not disclose data, findings, or decisions outside the scope of communication defined in this Charter. Materials provided to DMC for analysis are highly confidential and should not be disclosed in any way to unauthorized third parties
Will the IDMC be blinded to the treatment allocation	The DMC will not be blinded to the treatment allocation
To whom the DMC will communicate the decisions/ recommendations that are reached	DMC recommendations duly voted and approved are transmitted in writing or by teleconference from the President of the DMC to the Academic Steering committee expeditiously, at the latest within the established deadline
**Proposal of the statistical analysis plan**	
General principles and interim analyses	There will be two pre-planned interim analyses for safety and efficacy evaluation, after 96 patients and 234 of patients with the complete follow up to the primary outcome. Since the recruitment rate for the study is expected to increase giving the increase in number of cases of COVID-19 in Brazil it is possible that by the time all the patients for the second interim analysis had completed the follow-up for the primary outcome, the entire sample has already been recruited. Therefore, in this specific situation, the second interim analysis will be cancelled.<br>We will use the Haybittle-Peto boundary stopping rule for both safety and efficacy based on the evidence of significant differences between intervention or control group regarding ventilator free days at day 28, mortality or adverse events. The stopping rule for safety will be a p-value <0.01 and for efficacy p-value <0.001. The Haybittle-Peto boundary is a conservative stopping rule at interim analysis that has minimal impact in increasing type I error in two-arm trials
Primary outcome analysis and All-cause mortality analysis	The locking of the database will be performed after obtaining 28 days of follow-up of all patients and all the necessary actions to obtain follow-up are performed. The main analysis will be made considering the intention to treat principle<br>The primary outcome is to evaluate the effectiveness of early intravenous (IV) dexamethasone administration in ventilator-free days at 28 days after randomization, defined as alive and free from mechanical ventilation in adult patients with moderate or severe ARDS due to confirmed or probable SARS-CoV-2 infection. Patients discharged from the hospital alive before 28 days will be considered alive and free from mechanical ventilation at day 28. Number of days free from mechanical ventilation will be presented as mean and standard deviation. The treatment effect will be presented as mean difference, with 95% confidence interval and P-value. We will use a generalized linear model with beta-binomial distribution or zero/one inflated beta distribution, with center as random effect and adjusted for age, corticosteroid use before randomization and PaO_2/FiO_2 ratio<br>All-cause mortality rates at 28 days will be analyzed using a mixed Cox model, with centers as random effects (frailty model)
Safety and stopping standards	If there is a general increase in severe adverse events at 28 days with a two-tailed alpha threshold <0.01, the DMC will consider efficacy data together with safety information to consider stopping the study, also the DMC can choose to wait for the next interim analysis for weighting. To do this, the DMC will have access to the entire study database required for this specific intermediate analysis and may request additional data if necessary. If the study is not interrupted after any intermediate analysis, the alpha thresholds for severe adverse events will not be adjusted in the final statistical analysis. The occurrence of other non-severe adverse events (hyperglycemia) will also be weighted by DMC<br>Additionally, the DMC will consider other factors outside the rigid limits mentioned above to prepare a recommendation on the study. Safety and efficacy findings often need to be weighed along with external evidence outside the rigid limits. We believe that the DMC is free to carry out such consideration and provide its opinion in these terms
**Decision making**	
What decisions/ recommendations will be open to the DMC	DMC will recommend one of the following written actions to the Academic Steering Committee:<br>1. Continue the study according to the protocol and any related changes<br>2. Modify the study protocol. Modifications may include, but are not with others, changes in inclusion/exclusion criteria, frequency of safety monitoring, changes in study procedures<br>3. Pause inclusion, with pending resolution of a specified problem<br>4. Interrupt the study
How decisions or recommendations will be reached within the DMC	DMC members formally vote on all recommendations to be submitted to the steering committee. To vote, a Member of the DMC must be present at the meetings convened. A simple majority vote of the members transmits a proposal, motion or recommendation to the Academic Steering Committee
**Reporting**	
To whom will the DMC report their recommendations/ decisions, and in what form	The DMC will report their recommendations/decisions to the Academic Steering Committee through a letter or e-mail within one week after the DMC meeting. A copy of the DMC recommendation will be stored in the trial master file

Agreement and potential competing interests form

COVID-19-associated ARDS treated with DEXamethasone: CoDEX Trial

Please complete the following document and return to the CoDEX Trial Coordinator.

☐ I have read and understood the DMC Charter version 1.1, dated June 10th, 2020.
☐ I agree to join the DMC for this trial.
☐ I agree to treat all sensitive trial data and discussions confidentially.

The avoidance of any perception that members of a DMC may be biased in some fashion is important for the credibility of the decisions made by the DMC and for the integrity of the trial.

Possible competing interest should be disclosed to the trial Steering Committee. In many cases simple disclosure up front should be sufficient. Otherwise, the (potential) DMC member should remove the conflict or stop participating in the DMC.

☐ **No,** I have no competing interests to declare.
☐ **Yes,** I have competing interests to declare (please detail below).

Please provide details of any competing interests:

____

____

Name: ____________________________

Signed: ____________________________ Date: _______________
